# Impact of ASA-score, age and learning curve on early outcome in the initiation phase of an oncological robotic colorectal program

**DOI:** 10.1038/s41598-020-72025-3

**Published:** 2020-09-15

**Authors:** Hülya Sarikaya, Tahar Benhidjeb, Sergiu I. Iosivan, Theodoros Kolokotronis, Christine Förster, Stephan Eckert, Ludwig Wilkens, Alaa Nasser, Sebastian Rehberg, Martin Krüger, Jan Schulte am Esch

**Affiliations:** 1 Department of General and Visceral Surgery, Evangelisches Klinikum Bethel, Schildescher Str. 99, 33611 Bielefeld, Germany; 2 Institute of Pathology, Clinic Region Hannover, Hannover, Germany; 3 Department of Anaesthesiology, Intensive Care-, Emergency- and Pain-Medicine, Evangelisches Klinikum Bethel, Bielefeld, Germany; 4 Department of Gastroenterology and Internal Medicine, Evangelisches Klinikum Bethel, Bielefeld, Germany; 5 The New European Surgical Academy, Berlin, Germany

**Keywords:** Cancer therapy, Surgical oncology, Colorectal cancer

## Abstract

The ASA score is known to be an independent predictor of complications and mortality following colorectal surgery. We evaluated early outcome in the initiation phase of a robotic oncological colorectal resection program in dependence of comorbidity and learning curve. 43 consecutive colorectal cancer patients (median age: 74 years) who underwent robotic surgery were firstly analysed defined by physical status (group A = ASA1 + 2; group B = ASA3). Secondly, outcome was evaluated relating to surgery date (group E: early phase; group L: late phase). There were no differences among groups A and B with regard to gender, BMI, skin-to-skin operative times (STS), N- and M-status, hospital-stay as well as overall rate of complications according to Dindo-Clavien and no one-year mortality. GroupA when compared to group B demonstrated significantly lower mean age (65.5 years ± 11.4 years vs 75.8 years  ± 8.9 years), T-stage and ICU-stay. When separately analyzed for patients age ICU-stay was comparable (> 75 years vs. < 75 years). Group E and L demonstrated comparable characteristics and early outcome except more frequent lymphatic fistulas in group E. STS was reduced in group L compared to group E. Beyond learning curve aspects in our series, we could demonstrate that patient’s physical condition according to ASA rather than age may have an impact on early outcome in the initial phase of a robotic oncological colorectal program.

## Introduction

The American Society of Anesthesiologists classification of physical condition (ASA) is a well-accepted, strong predictor of post-operative medical complications and mortality following surgical procedures^1^. ASA3 or higher represents an independent risk factor for survival in colorectal surgery^2^. Subsequent to laparoscopic oncological colorectal surgery, ASA3 patients were demonstrated to experience a higher rate of postoperative 30-day complications, 30-day mortality and complications stratified according to the Clavien-Dindo classification^3^ when compared to ASA status 1 or 2^4^.

Age is not an independent factor for complications after colorectal surgery. However, ASA-score seems to be higher with age^5^. This is noteworthy in the light of a worldwide demographic change towards an ageing community, healthcare systems are increasingly facing medical, ethical, and economical challenges in treating geriatric patients. This includes oncological diseases since cancer incidence increases with age^6^. Robotic colorectal surgery (RCS) as the evolution of minimal invasive surgery, may improve on the advantages of laparoscopic procedures, and as such represents an interesting therapeutic option for the senior population^7,8^. However, some concerns were postulated in the use of robotic-assisted surgery in older patients, especially longer operation time and increased physical stress due to table positioning during the surgical procedure^9,10^. Concerning the impact of age on postoperative outcome after major colorectal surgery discussion remains controversial^11^. However, rather than age alone pre-existing comorbidity seems to be an important predictor for the outcome following colorectal surgery in elderly patients^12^.

The learning curve of an RCS program is characterised by factors like surgical procedure time^13,14^. Training and tutor-support demonstrated satisfying safety levels with initiation of robotic colorectal programs^15^. However, data on the impact of patients’ co-morbidity on the safety of an initiation phase of a robotic oncological colorectal surgery program are widely missing. In this retrospective study, we present our early experience of the initial 43 consecutive cases of oncological RCS that were stratified for ASA-score. We further analysed characteristics of patients, surgery, oncology and early outcome when stratified for phase of the resection program and for patient’s age in order to evaluate the role of patient’s pre-surgical co-morbidity and physical condition for safety and outcome in the initiation phase of oncological RCS.

## Results

### Characteristics and early outcome in dependence of patients’ physical condition and age

To evaluate pre-existing co-morbidity stratification for ASA revealed Group A representing 46.5% (n = 20) and group B 53.5% (n = 23) of the analysed cohort. Overall mean age of patients in this study was 71.0 years ± 11.29 years (range 44.0 years to 90.0 years). In group A, age ranged significantly lower compared to group B with 65.5 years ± 11.4 years and 75.8 years ± 8.9 years respectively (p = 0.002). Gender and BMI were comparable between groups (Table 1). The distribution of tumor localization and thereby resulting resected part of the colo-rectum was comparable among groups. Likewise, no differences were observed among the two groups concerning phase of the RCS program, rate and type of primary colostomies, frequency of emergency procedures, operative time (skin-to-skin; overall mean 298.7 ± 67.7  min), rate of planned hybrid procedures and unplanned conversion to open and time to first bowel movement (Table 1).

**Table 1:** Patients’ and surgical characteristics.

Variables	Group A: ASA 1 + 2 n = 20 (2 + 18)	Group B: ASA 3 n = 23	p-value
**Patient characteristics**			
Gender (male:female (% (n))	35.0:65.0 (7:13)	39.1:60.9 (9:14)	0.780
Age (mean ± SD)	65.5 ± 11.4	75.8 ± 8.9	**0.002**
BMI (mean ± SD)	26.1 ± 3.8	25.9 ± 4.6	0.857
**Surgical characteristics**			
Phase of robotic colorectal program			0.571
Early phase (2016/2017; (% (n))	35.0 (7)	43.5 (10)	
Late phase (2017/2018; (% (n))	65.0 (13)	56.5 (13)	
Primary colostomy			0.538
None (% (n))	85.0 (17)	82.6 (19)	
Protective temporary loop (% (n))	5.0 (1)	13.0 (3)	
Permanent ending (% (n))	10.0 (2)	4.4 (1)	
Tumor localization/ resected structure			0.695
Right colon (% (n))	65.0 (13)	52.2 (12)	
Left colon/sigmoid (% (n))	10.0 (2)	13.0 (3)	
Rectum (% (n))	25.0 (5)	34.8 (8)	
Emergency procedure	0.0 (0)	4.4 (1)	0.345
Operative time (min) (skin-to-skin, mean ± SD)	305.3 ± 78.4	293.0 ± 58.0	0.560
Unplanned conversion to open rate (% (n))	0.0 (0)	4.4 (1)	0.345
First bowel movement (days) (mean ± SD)	1.15 ± 1.18	1.56 ± 1.53	0.363

Bold marks significants (p < 0.05).

*SD* standard deviation of the mean.

Concerning initial staging from an oncological point of view group B-patients demonstrated initially with locally more advanced malignancies represented by a T0–T4-status distribution more pronounced towards higher stages in group B if contrasted to group A (p = 0.005; Table 2). Nodal disease and distant metastases as well as rate of neo-adjuvant therapy were equally distributed among groups A and B. The trend in more advanced LN-yield in group A compared to group B was not significant (31.1 ± 17.8 and 23.8 ± 11.4 respectively; p = 0.117). All surgical procedures included in this study resulted in locally tumor negative resection margins (R0).

**Table 2:** Patients’ early outcome and oncological characteristics.

Variables	Group A: ASA 1 + 2 n = 20 (2 + 18)	Group B: ASA 3 n = 23	p-value
**Oncological characteristics**			
Neoadjuvant therapy (% (n))	15.0 (3)	8.7 (2)	0.520
T-stage			**0.005**
0 (% (n))	5.0 (1)	0.0 (0)	
1 (% (n))	40 (8)	0.0 (2)	
2 (% (n))	10.0 (2)	17.4 (4)	
3 (% (n))	30.0 (6)	73.9 (17)	
4 (% (n))	15.0 (3)	8.7 (2)	
T-stage (0–2:3–4 (% (n))	55.0:45.0 (11:9)	17.4:82.6 (4:9)	**0.010**
N-stage			0.256
0 (% (n))	75.0 (15)	56.5 (13)	
1 (% (n))	10.0 (2)	30.4 (7)	
2 (% (n))	15.0 (3)	13.0 (3)	
M-stage (0:1 (% (n))	95.0:5.0 (19:1)	82.6:17:4 (19:4)	0.206
Lymph node harvest (mean ± SD)	31.1 ± 17.8	23.8 ± 11.4	0.117
Tumor negative resection margin (local R0)	100 (20)	100 (23)	1,0
**Early outcome**			
ICU stay in days (median (max/min))	0 (0/25)	1 (0/48)	**0.023**
Hospital stay in days (mean ± SD)	12.1 ± 5.6	17.0 ± 13.96	0.175
Readmission within 6 months (% (n))	0.0 (0)	4.4 (1)	0.345
Redo surgery within 30 days (% (n))	0.0 (0)	4.4 (1)	0.345
1-year mortality (% (n))	0.0 (0)	0.0 (0)	1.000
Complications (Clavien-Dindo (CD) classification)			0.172
CD 0 (% (n))	60.0 (12)	43.5 (10)	
CD I (% (n))	40.0 (8)	34.8 (8)	
CD II (% (n))	0.0 (0)	17.4 (4)	
CD III (% (n))	0.0 (0)	4.4 (1*)	
Blood transfusion (% (n))	0.0 (0)	13.0 (3)	***0.09***
Wound infection (% (n))	15.0 (3)	17.4 (4)	0.832
Anastomotic leak (% (n))	0.0 (0)	4.4 (1)	0.345
Lymphatic fistula (% (n))	10.0 (2)	13.0 (3)	0.756
Urinary bladder infection (% (n))	15.0 (3)	30.4 (7)	0.232
Pneumonia (% (n))	0.0 (0)	17.4 (4)	***0.050***
Incisional hernia (% (n))	0.0 (0)	8.7 (2)	0.177

Bold marks significants (p < 0.05), bold/italic marks a trend (p > 0.05 and < 0.1).

*SD* standard deviation of the mean.

*CD-IIIa.

In this study, no 1-year mortality was observed. Overall, there were no significant differences between the two groups with regard to rate or severity of complications according to Clavien-Dindo, as well as the conversion rates or the need for placement of an intestinal stoma (Table 2). One patient in group B experienced an anastomotic leakage following low rectal resection subsequent to neoadjuvant chemoradiotherapy as the only CD-type-III complication in this study managed by redo-surgery performing a Hartmann procedure on post-surgery day 6. The four pneumonias and three necessities for blood transfusions were solely observed for ASA3 patients which was statistically a non-significant trend. Indication for blood transfusion originated in a pre-interventional manifestation of anaemia plus simultaneous cardiac co-morbidity. Blood loss did not exceed 400 ml. Frequency of wound infections, lymphatic fistulations and urinary bladder infections were comparable between groups A and B. Lymphatic fistulas were all but one subsequent to CME, mild, transient and ceased subsequent to medium-chain fatty acid diet for maximum 4 days. Hospital stay, and number of readmissions within 6 months and redo surgery within 30 days were also comparable between groups A and B (Table 2). However, ICU-stay was significantly prolonged on average in group B (median/mean 0.0/1.6 days; min/max: 0.0/25.0 days) as compared to group A (median/mean: 1.0/2.54 days; min/max: 0.0/48.0 days; p = 0.023).

Interestingly, in separate analyses of the same cohort stratified for age up to 75 years of age (n = 26) compared to older than 75 years (n = 17) ICU-stay was comparable among these two age groups (median/mean: 0.0/4.2 days; min/max: 0.0/25.0 days vs. median/mean: 0.0/3.7 days; min/max: 0.0/48.0 days; p = 0.901). Furthermore, with this approach of age-related analyses distribution of cancer location (p = 0.603), complication profiles according to the DC-classification (p = 0.789), pneumonia (p = 0.128) and T-stage (p = 0.543) were comparable between age groups (data not shown).

### Early vs. late phase of the initial experience in robotic oncological colorectal surgery

To evaluate effects of a learning curve on early outcome, an analogue analysis of characteristics and variables was performed on 17 procedures performed in the early phase (group E: first 15 months) and the 26 surgeries in the later phase of our RCS program (group E: second 15 months). Patients’ characteristics like gender, age, distribution of the involved colorectal part as well as ASA-scores were comparable between group E and group L (Table 3). The BMI demonstrated a trend to be increased in group L compared to group E. Nevertheless, the average STS was reduced by 43 min in group L in contrast to group E (p = 0.041). Hospital- and ICU-stay did not show differences among groups E and L.

**Table 3:** Early vs. late phase of the oncological robotic colorectal surgery program.

Variables	Group E: Early phase n = 17	Group L: Late phase n = 26	p-value
**Patient and surgical characteristics**			
Sex/m:f (% (n))	35.3 : 64.7 (6:11)	38.5 : 61.5 (10:16)	0.834
Age (mean ± SD)	70.2 ± 12.4	71.6 ± 10.8	0.696
BMI (mean ± SD)	24.5 ± 3.3	26.9 ± 4.5	***0.063***
ASA 1–2:3 (% (n))	41.2:58.8 (7:10)	50.0:50.0 (13:13)	0.571
ASA score			0.767
1 (% (n))	5.9 (1)	4.2 (1)	
2 (% (n))	35.3 (6)	41.7 (12)	
3 (% (n))	16.1 (10)	54.2 (13)	
**Resected colorectal part**			0.134
Right colon (% (n))	41.2 (7)	69.2 (18)	
Left colon/sigmoid (% (n))	11.8 (2)	11.5 (3)	
Rectum (% (n))	47.1 (8)	30.2 (5)	
Emergency procedure	3.2 (1)	8.3 (2)	0.454
Operative time in min (skin-to-skin, mean ± SD)	324.6 ± 87.0	281.8 ± 45.7	**0.041**
ICU stay in days (median (max/min))	0 (0/25)	0 (0/48)	0.967
Hospital stay in days (mean ± SD)	16.6 ± 17.1	13.4 ± 6.3	0.394
**Oncological characteristics and outcome**			
T-stage (0–2:3–4 (% (n))	41.2:58.8 (7:10)	30.8:69.2 (8:18)	0.484
N-stage			0.465
N0 (% (n))	70.6 (12)	61.5 (16)	
N1 (% (n))	11.8 (2)	26.9 (7)	
N2 (% (n))	17.7 (3)	11.5 (3)	
M1-stage (% (n))	5.9 (1)	15.4 (4)	0.342
LN harvest over all (mean ± SD)	20.2 ± 10.4	31.8 ± 15.9	**0.011**
LN harvest right colon (mean ± SD)	16.3 ± 8.5 (n = 7)	35.8 ± 16.9 (n = 18)	**0.008**
LN harvest left colon/sigmoid (mean ± SD)	18.0 ± 4.2 (n = 2)	23.0 ± 11.1 (n = 3)	N/A
LN harvest (rectum)	24.2 ± 12.2 (n = 8)	22.4 ± 8.7 (n = 5)	0.395
Neo-adjuvant therapy (% (n))	11.8 (2)	11.5 (3)	0.982
**Patient outcome**			
Complications (Clavien-Dindo (CD) classification)			0.411
CD 0 (% (n))	47.1 (8)	53.9 (14)	
CD I (% (n))	35.3 (6)	38.5 (10)	
CD II (% (n))	17.7 (3)	3.9 (1)	
CD III (% (n))	0.0 (0)	3.9 (1*)	
Blood transfusion	5.9 (1)	7.7 (2)	0.820
Redo surgery within 30 days (% (n))	0.0 (0)	3.9 (1)	0.413
Rate of anastomotic leak (% (n))	0.0 (0)	3.9 (1)	0.413
Lymph fistula (% (n))	23.5 (4)	3.9 (1)	**0.049**
Incisional hernia (% (n))	11.8 (2)	0 (0)	***0.073***

Bold marks significants (p<0.05), bold/italic marks a trend (p > 0.05 and < 0.1).

*SD* standard deviation of the mean; early phase: 5/2016-5/2017; late phase: 6/2017-8/2018.

*CD-IIIa.

Patients in the two phases of our program demonstrated equal distributions of oncological aspects like staging (TNM) and neo-adjuvant therapy. Interestingly, the LN harvest was superior in group L if contrasted to group E which was mainly attributable to the yield with right colectomy procedures in this analysis (Table 3). The overall distribution of complications according to the DC-classification was comparable as were specifically rates of pneumonia and blood transfusions. However, it is noteworthy, that the frequency of lymphatic fistulas in the first phase of the robotic program could be significantly reduced (Table 3). These were all managed by temporary dietary modification. Further, we experienced two trocar hernias in the first phase.

## Discussion

Comorbidity that positively correlates with increasing age is more likely to represent the decisive factor for postoperative results in general and specifically subsequent to colorectal resections than patients’ age alone^12,16^. This hypothesis was supported by data in this study taken from the initiation phase of our RCS program demonstrating patients’ ASA-scores rather than age at time of oncological colorectal resection to correlate with duration of post-surgical ICU-stay and rate of pneumonia.

Laparoscopic surgery for colorectal cancer provides several advantages in comparison with open surgery especially in regard to less postoperative pain, less blood loss, faster return to prior activities and lower healthcare costs^17^. Recent studies underlined the potential of laparoscopic surgery to be performed safely on both older and younger patients with no differences compared with open surgery in respect to morbidity, length of hospital stay and better long-term outcome^18,19^. Up to now, studies could not demonstrate that robotic surgery is superior to conventional laparoscopy with regard to resection for rectal cancer^20–22^. A few studies comparing robotic to open surgery revealed reduced blood loss, lower morbidity and shorter hospital stay, but increased overall operation time and costs associated with robotic surgery^23,24^. Reports on outcome in dependency of pre-surgical comorbidity especially in the initiation phase of RCS programs are scarce to date. The present study supports oncological RCS is feasible and safe in the pronounced comorbid population with results in ASA3 individuals comparable to ASA1/2 patients. Further with comparable adverse event intensity profiles according to Dindo-Clavien independent of the age groups, our data support the perspective that advanced age on its own should not be regarded as a risk factor for higher rates of morbidity subsequent to RCS, as stated by others for colorectal surgery in general^11^. We experienced only one anastomotic leak, one unplanned conversion to open surgery in a T4-carcinoma of the hepatic flexure due to duodenal infiltration and no mortality during the 12-months follow-up period. These favourable results may reflect the significance of interdisciplinary perioperative management of patients with pronounced comorbidities as implemented in our program.

Operating time seems rather high in this series^25–27^. However, mean skin-to-skin time ranged within those reported previously for the early experience with adopting RCS^28–30^**.** As expected, there was a significant reduction of operation times towards the second phase of this program at our institution as observed by others representing an important aspect of a learning curve. Further, we were able to improve the lymph node yield in the second phase due to a technical modification of CME in the course of right colectomy as reported previously^31,32^. A second technical aspect of improvement of our program was the implementation of fascial sutures of all trocar incisions—a measure that resulted in a lack of further trocar related herniations, as reported by others with robotic surgery of the abdomen^33^. Despite prolonged operation time in comparison to open colorectal surgery, no reports to date revealed a significant effect upon postoperative morbidity. In our study, prolonged operation time did not have an impact on postoperative morbidity.

We are aware of the limitations of our study including some heterogeneity of the investigated patients with regard to group size, as well as the lack of a laparoscopic or open control group. Moreover, some complications might be due to neo-adjuvant treatment rather than age alone. However, the purpose of this study was to evaluate the impact of age, ASA-score and learning curve on the outcome of an initiation phase of a single center RCS program. Therefore, limitations as mentioned were by their very nature not completely preventable. Noteworthy, others reported data on initial robotic surgical experience on similar numbers of patients when compared to our numbers in the early and late phase of our program with a comparable size definition of phase 1 and phase 2^34–36^. Multicentric evaluation of the initiation phase would be favorable, however complex to implement.

We demonstrated aspects of a learning curve with improvement of operating time and technical aspects with implementing RCS over time without significant differences in the overall complication rate and severity when comparing the early and late phase of the program initiation. Further, this study supports the hypotheses that post-surgical complication profiles and early outcome correlate with pre-existing comorbidity rather than patients’ age. Nevertheless, ASA3 patients although bearing a pronounced necessity for perioperative high care management can safely be included in the initiation phase of an oncological RCS program with early results comparable to patients with more preserved physical condition. Long-term studies in larger patients´ cohorts are required to further evaluate comorbidity and patient condition scores like ASA as predictor for clinical and oncological outcome in RCS.

## Methods

### Study design and robotic system

We conducted this retrospective study with approval of the local ethics committee (Heinrich-Heine-University, Duesseldorf, Germany; study-no.: 2018-229-RetroDEuA). Informed consent was not necessary according to local ethical regulations in a retrospective investigation as performed here (ethics committee of the Heinrich-Heine-University Düsseldorf, Germany (study 2018-229-RetroDEuA)). All the experimental protocols for involving human data in the study were in accordance to guidelines of the Declaration of Helsinki (64th WMA General Assembly, Fortaleza, Brazil, October 2013). 66 consecutive patients underwent colorectal procedures at our center between the initiation of the program in May 2016 and August 2018. 43 of those were pathologically diagnosed with colorectal cancer and included in this study without exclusion. Surgery was performed by the same surgeon (J.S.a.E.) with the DaVinci Xi system (Intuitive Surgical, Aubonne, Switzerland) which is connected to a TruSystem 7000dV OR-table (TRUMPF Medicine System, Saalfeld, Germany) enabling integrated table motion without the need to detach the robotic device.

### Surgery

Pneumoperitoneum was set to a pressure of 10 to 12 mm Hg. For right hemicolectomy, we used the suprapubic robotic trocar setup, positioning the 4 ports along a horizontal line 3–5 cm above the pubis with a spacing of 7–8 cm plus 1 OR-table assistant operated 13 mm-trocar in the left lateral abdomen as reported previously^31,32^. These patients were positioned in a 23° to 25° head down and 12° to 14° left sided orientation of the OR-table that provides an optimal position for retro-colic dissection as well as superior-mesenteric vessel-development with central vascular ligation. For anterior rectal resection, we utilized the upper right access, positioning the 4 ports along a line from right rib arch to anterior superior iliac spine with 7–8 cm distance between the ports. The standardized medial-to-lateral approach with splenic flexure take down was used for complete colonic mobilization to perform the pelvic anastomosis. Nerve sparing total mesorectal excision (TME) was performed according to Heald’s described principles^37^. All colo-colostomies and Ileo-colostomies respectively were performed extracorporeally side to side. For Colo-rectostomies the anvil was inserted upfront via a mini laparotomy that was used simultaneously for specimen extraction to prepare for circular stapler anastomoses. The circular anastomoses was completed by its nature intra-corporeal. Deep rectal anastomoses were secured by a protective ileostomy.

### Study groups and parameters

This study was designed to focus on short-term postoperative outcome. In order to evaluate the ASA dependency of early outcome, patients were divided in 2 groups (group A: ASA 1 or 2; group B: ASA ≥ 3). In a second analysis, patients were divided in 2 groups according to date of surgery representing the early phase (group E: 5/2016-5/2017) and the late phase (group L: 6/2017-8/2018) of our RCS program development in order to investigate the safety of the procedure with respect to the learning curve.

Parameters analysed to characterise patient’s and surgical aspects included gender, age, BMI, rate of primary colostomies, tumor localization, frequency of emergency procedures, operative time (skin-to-skin), rate of planned hybrid procedures and unplanned conversion to open, time to first bowel movement. Oncological characteristics encompassed rate of patients with neoadjuvant therapy, staging parameters like T-, N- and M- stage as well as mean numbers of harvested lymph nodes (LN). Parameters evaluated to characterise outcome included intensive care unit and hospital stay, frequency of readmission within 6 days and redo surgery within 30 days, 1-year mortality, rate of complications according to Clavien-Dindo-classification (CD)^3^, necessity of blood transfusion, rate of wound infections, anastomotic leakage, lymphatic fistulas, urinary bladder infections, pneumonia and incisional hernias.

### Statistics

Statistical analysis was performed with STATA 15.1 Software (StataCorp Stata MP 15.1 USA). For two group comparison (early vs. late phase) for proportional variables, two-sided student t-test was used for normal distributed values and Wilcoxon Rank Sum Test for non-normal distributed values. Pearson-Chi-Square test was performed for categorical variables. P-value of < 0.05 were regarded to be significant.

## Data Availability

The datasets generated during and/or analysed during the current study are available from the corresponding author on reasonable request.
